# Development of a Short Telomere Zebrafish Model for Accelerated Aging Research and Antiaging Drug Screening

**DOI:** 10.1111/acel.70007

**Published:** 2025-02-08

**Authors:** David Hernández‐Silva, María D. López‐Abellán, Francisco J. Martínez‐Navarro, Jesús García‐Castillo, María L. Cayuela, Francisca Alcaraz‐Pérez

**Affiliations:** ^1^ Grupo de Telomerasa, Cáncer y Envejecimiento Hospital Clínico Universitario Virgen de la Arrixaca Murcia Spain; ^2^ Instituto Murciano de Investigación Biosanitaria‐Pascual Parrilla (IMIB‐Pascual Parrilla) Murcia Spain; ^3^ Universidad Católica de Murcia (UCAM) Murcia Spain; ^4^ Centro de Investigación Biomédica en Red de Enfermedades Raras (CIBERER) Madrid Spain

**Keywords:** antiaging screening, premature aging, short telomeres, zebrafish

## Abstract

Increased life expectancy is associated with a higher risk of age‐related diseases, which represent a major public health challenge. Animal models play a crucial role in aging research, enabling the study of diseases at the organism level and facilitating drug development and repurposing. Among these models, zebrafish stands out as an excellent in vivo system due to its unique characteristics. However, the longevity of zebrafish is a limitation for research, as it often takes too long to obtain results within a reasonable timeframe. To address this, we have developed a short telomere zebrafish line (ST2) with a premature aging phenotype during the larval stage. Although less extreme than the tert‐deficient G2 larvae, ST2 larvae exhibit reduced telomerase expression and activity, along with shortened telomeres. they also exhibit increased cellular senescence, apoptosis, and premature death. As a proof of concept, we evaluated the antiaging effects of two compounds: resveratrol (a polyphenol) and navitoclax (a senolytic). Our results confirm the antiaging properties of resveratrol, which improves telomere maintenance. However, navitoclax does not attenuate the ST2 phenotype. Taking advantage of the zebrafish larval model, this premature aging system provides a valuable platform for in vivo testing of rejuvenating molecules through drug screening, using telomere length or survival as a readout.

## Graphical abstract

Larvae derived from a cross between tert deficient and haploinsufficient fish show a premature aging phenotype, characterized by telomere attrition and early death. The ST2 larval model is a valuable tool for rapid screening of antiaging molecules. Given that organogenesis occurs within the first 48 h, this model also allows for the conduction of aging studies in a significantly shortened time frame.

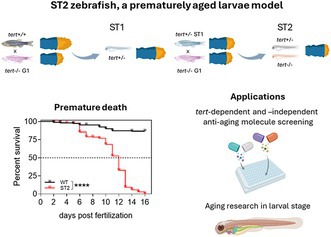

**Abbreviations**
AO: acridine orange
DC: dyskeratosis congenita
DDR: DNA damage response
G1: generation 1
qPCR: quantitative polymerase chain reaction
qTRAP: quantitative telomerase repeated amplification protocol
ROI: region of interest
ROS: reactive oxygen species
RTA: relative telomerase activity
RT‐qPCR: real‐time quantitative polymerase chain reaction
SASP: senescence‐associated secretory phenotype
SA‐β‐gal: senescence‐associated β‐galactosidase
SIPS: stress‐induced premature senescence
ST2: short telomere 2nd round
TERT: telomerase reverse transcriptase
TR: telomerase RNA
WT: wild type
ZFIN: zebrafish information network

## 1 Introduction

Aging is a biological process that occurs in eukaryotic organisms. It consists of a gradual and generalized decline in performance as a result of the accumulation of damage to molecules, cells, and tissues over a lifetime, with an increased risk of many diseases (Rodriguez‐Rodero et al. 2011). At the cellular level, one of the best understood molecular mechanisms responsible for aging is the progressive attrition of telomeres that occurs with each cell division (Harley et al. 1990). Telomeres consist of simple repetitive sequences whose length is maintained by telomerase. Telomerase is a ribonucleoprotein with two essential components: the telomerase reverse transcriptase (TERT) and telomerase RNA (TR), which provides the template for the reverse transcription of new telomere DNA by TERT (Blackburn 1991). When the telomere length becomes critically short, chromosomal instability occurs and then the cell enters a state known as replicative senescence (Lopez‐Otin et al. 2023).

Senescence is characterized by a permanent cell cycle arrest and the acquisition of a senescence‐associated secretory phenotype (SASP) (Hernandez‐Segura et al. 2018). On the other hand, other genetic and environmental factors, such as (i) activation of oncogenes, (ii) inhibition of tumor suppressor genes, (iii) accumulation of DNA damage, and (iv) presence of reactive oxygen species (ROS), can contribute to aging through another type of senescence called stress‐induced premature senescence (SIPS) (de Magalhaes and Passos 2018).

Cellular senescence is necessary for many physiological processes, such as tissue remodeling during development and, paradoxically, tissue growth after injury. However, the chronic accumulation of senescent cells can negatively affect tissue regeneration and function, making tissues more susceptible to inflammation and age‐related diseases (Munoz‐Espin and Serrano 2014).

Nowadays, with increased life expectancy, preventing of the progression of aging and curing age‐related diseases has become the goal of many researchers. Telomerase activation has emerged as a potential solution to mitigate this phenotype. Indeed, numerous studies using inducible animal models have demonstrated its efficacy. For instance, Jaskelioff and colleagues showed that several aging phenotypes in a mouse model of accelerated telomere loss could be reversed within 4 weeks of telomerase reactivation (Jaskelioff et al. 2011). This discovery suggests a promising therapeutic avenue whereby telomerase activation could rescue short telomeres present in telomere syndromes and age‐related diseases, thereby promoting tissue homeostasis and mitigating associated pathologies. Gene therapy studies using adeno‐associated virus (AAV) or cytomegalovirus (CMV) vectors carrying *Tert* have shown that increased Tert induction correlates with improved quality and lifespan (Bar et al. 2016; Bernardes de Jesus et al. 2012; Jaijyan et al. 2022). The human *hTERT‐Rluc* reporter transgene in adult mouse ear fibroblasts provides a useful in vitro tool for preclinical drug screening (Jia et al. 2011). Thus, compounds targeting this pathway may serve as effective strategies for rejuvenation, combating cellular senescence and mitigating its consequences (Shim et al. 2024). In addition to telomerase reactivation, antiaging strategies include a range of dietary and pharmacological interventions. Several approaches have been explored to slow down aging, including fasting (de Cabo and Mattson 2019), caloric restriction (Pifferi and Aujard 2019), exercise (Galloza et al. 2017), nutraceuticals (Shen et al. 2017), and senolytics (Kirkland and Tchkonia 2020).

To study aging with an in vivo model, two critical factors must be considered for the success of the research: the aging phenotype and the lifespan, the latter being the most limiting as it is usually too long to obtain results within a reasonable time frame (Mitchell et al. 2015). In recent decades, the zebrafish ( *Danio rerio* ) has positioned itself as an excellent in vivo model to study a variety of physiological and pathological processes, including aging (Cayuela et al. 2018; Lieschke and Currie 2007; Santoriello and Zon 2012). Unlike the mouse model, zebrafish have telomere lengths similar to humans and retain the same telomere maintenance mechanisms (Anchelin et al. 2011; Henriques and Ferreira 2012). On the other hand, gene editing tools are highly developed and the transparency and small size of embryos and larvae allow for large‐scale fluorescence in vivo assays and drug screening (Patton et al. 2021). Several studies on telomerase and telomere length in zebrafish have already shown that they are useful markers for assessing the aging process (Anchelin et al. 2011; Carneiro, de Castro, et al. 2016; Carneiro, Henriques, et al. 2016). The aging phenotype of zebrafish has been well described using a model of premature aging, the telomerase‐deficient zebrafish line (*tert* ^−/−^). The phenotype included lack of telomerase activity, telomere shortening, chromosomal instability, persistent reduction in cell proliferation, acute apoptotic response, accumulation of DNA damage response (DDR), senescence, and, finally, tissue atrophy (Anchelin et al. 2013; Henriques et al. 2013). However, the lifespan of current aging zebrafish models (*tert* ^−/−^ G1) is still too long, while the second generation (*tert* ^−/−^ G2) is so severely affected that it is too short (Anchelin et al. 2013). Therefore, generating a rapidly aging zebrafish model that lives long enough to study the aging process at the larval stage is highly desirable for drug screening. In the present work, we introduce a crossing strategy to obtain a short telomere zebrafish line (hereafter ST2) which produces a prematurely aging zebrafish larval model to search for new antiaging treatments through drug or nutraceutical screening.

## 2 Materials and Methods

### 2.1 Study Approval

The experiments performed comply with the Guidelines of the European Union Council (Directive 2010/63/EU) and the Spanish RD 53/2013. The experiments and procedures were performed with the approval of the Bioethics Committee of the University Hospital Virgen de la Arrixaca (Spain).

### 2.2 Zebrafish Lines and Maintenance

**FIGURE 1:**
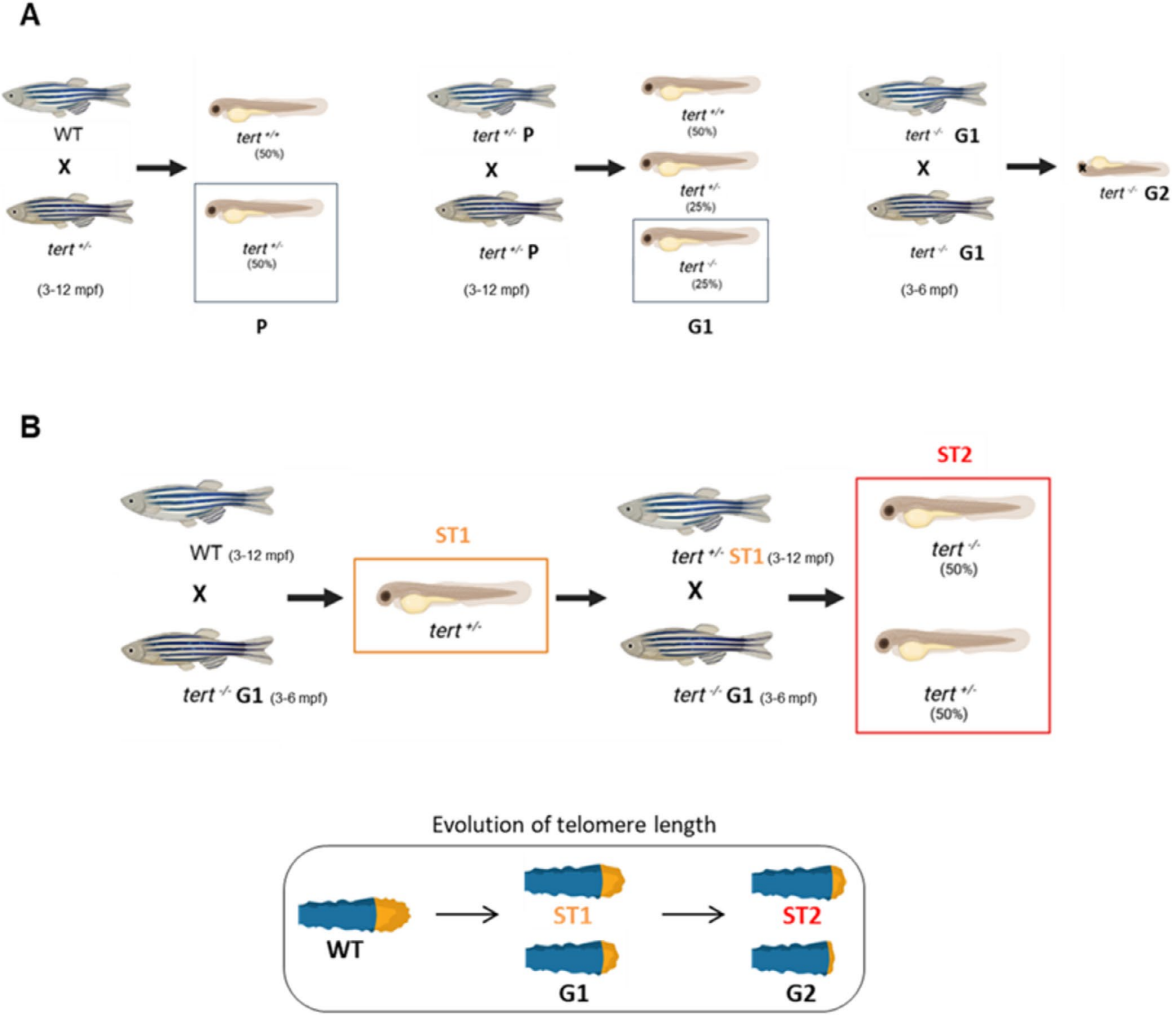
Workflow for the generation of the ST2 model. (A) Parental stocks of *tert* ^+/−^(P) zebrafish are generated by outcrosses to wild‐type (WT, *tert* ^+/+^) to then obtain the first‐generation progeny (G1) from a P heterozygous cross. (B) Generation of shortened telomere‐induced premature aging larvae (ST2) after two rounds of telomerase‐deficient zebrafish crosses, consisting of 50% *tert* ^+/−^ and 50% *tert* ^−/−^ fish. Telomere shortening of the ST2 line is less aggressive than that of the G2 line. The age range of the fish is indicated in parentheses.

Wild‐type AB zebrafish ( *Danio rerio* ) were obtained from the Zebrafish International Resource Centre and mated, staged, bred, and processed according to standard procedures. Details of husbandry and environmental conditions are available on protocols.io (DOI: dx.doi.org/10.17504/protocols.io.mrjc54n) (Widrick et al. 2018). The *tert*‐mutant line (allele hu3430) was obtained from the Sanger Institute and has been characterized previously (Anchelin et al. 2013; Henriques et al. 2013). The fish employed in the crosses exhibited a range of ages between three and 12 months for the wild‐type and *tert* ^+/−^ (both P and ST1), or between three and 6 months for the Tert‐deficient line (*tert* ^−/−^ G1), as illustrated in Figure 1.

### 2.3 *tert* Mutant Genotyping

The one‐month‐old juvenile fish were anesthetized with 0.05% benzocaine (Sigma) in order to obtain a biopsy from the apical region of the tail fin. The sample was incubated in alkaline lysis solution (NaOH 25 mM, EDTA 0.2 mM, pH 12) at 95°C for 20 min (Bruckner et al. 2022), cooled on ice, and subsequently neutralizing solution (Tris–HCl 40 mM, pH 5) was added. Following centrifugation, the supernatant was employed as a template for a PCR using the zf_*tert*Fwt forward primer and either the zf_*tert*Rwt (for the WT allele) or the zf_*tert*Rmut (for the mutant allele) reverse primers. Amplification protocol consisted in a denaturing step of 4 min at 94°C, followed by 40 cycles of 1 min at 94°C, annealing at 65°C for 30 s, and extension at 72°C for 30 s, and a final step of 10 min at 72°C. The sequence of the primers is provided in Table S1.

### 2.4 Analysis of Gene Expression

Total RNA was extracted from larvae at 3 days post‐fertilization (dpf) with *TRIzol* reagent (Thermo Fisher Scientific) using the *Direct‐zol RNA Miniprep Kits* (Zymo Research), and the first‐strand cDNA was synthesized using the *SuperScript VILO cDNA Synthesis Kit* (Invitrogen), according to the manufacturer's instructions. Real‐time PCR was performed on a StepOnePlus instrument (Applied Biosystems) using SYBR Premix Ex Taq (Perfect Real Time) (Takara). Reaction mixtures were incubated for 30 s at 95°C, followed by 40 cycles of 5 s at 95°C, 20 s at 60°C, and finally a melting curve protocol. Ribosomal protein S11 (*rps11*) was used to normalize zebrafish mRNA expression using the comparative Ct method ($2^{-\Delta C_{t}}$). The primers used are detailed in Table S1. In all cases, each PCR was performed with triplicate samples and repeated with three independent pools of 25 larvae.

### 2.5 Telomerase Activity Assay

Three independent pools of 25 zebrafish larvae were mechanically homogenized, and proteins were extracted using ice‐cold CHAPS lysis buffer (Millipore). Real‐time qTRAP was performed with 0.1 μg of protein extracts as previously described (Anchelin et al. 2011; Herbert et al. 2006). To confirm the specificity of the assay, a negative control treated with 1 μg of *RNase* at 37°C for 20 min was included for each sample (not shown). The standard curve obtained was *y* = 23.802–3.2295*x*. The data were collected and converted into relative telomerase activity units for calculation: RTA of sample = 10^(*C*t sample‐γint)/slope^.

### 2.6 Telomere Length Measurement

*gDNA* was obtained by using the *Wizard Genomic DNA Purification Kit* (Promega) following the manufacturer's instructions, and telomere length was measured by qPCR following the protocol described by (Cawthon 2002), using the primers detailed in Table S1, and 16 ng of *gDNA* as template. Real‐time PCR was performed on an ABI PRISM 7700 instrument (Applied Biosystems) using TB Green PCR Core Reagents (Applied Biosystems). Reaction mixtures were incubated at 95°C for 15 min, followed by 40 cycles of 95°C for 15 s, 54°C for 2 min, and finally 95°C for 15 s, 60°C for 1 min, and 95°C for 15 s. For each sample, the telomere content relative to the single‐copy ribosomal protein S11 (*rps11*) gene was calculated by using the comparative *C*_t_ method ($2^{-\Delta C_{t}}$). In all cases, each PCR was performed in triplicate.

### 2.7 Senescence Associated β‐Galactosidase (SA β‐Gal) Staining Assay

At 24 h post‐fertilization (hpf), larvae were treated with 1‐phenyl‐2‐thiourea (PTU) (3.8 μM) to prevent the formation of melanine. After 3 days posttreatment (dpt), the larvae were fixed in 4% paraformaldehyde (PFA) overnight at 4°C with stirring, then washed three times for 1 h in PBS (pH 7.4) and for a further 1 h in PBS (pH 6.0) at 4°C. Staining was performed overnight at 28°C with staining solution (5 mM potassium ferrocyanide, 5 mM potassium ferricyanide, MgCl_2_ 2 mM, and 1 mg/mL X‐gal in PBS adjusted to pH 6.0) with stirring. After staining, the larvae were transferred to a Petri dish with PBS (pH 7.0) and imaged using a Leica M150C magnifying glass equipped with a digital camera (DMC 5400, Leica). Quantification of SA‐β‐gal activity staining was performed using *ImageJ* software by calculating the percentage of blue area of each larva.

### 2.8 Reactive Oxygen Species (ROS) Staining

The level of oxidative stress in cells was determined by quantifying the release of H₂O₂ using the live cell fluorogenic substrate acetyl‐pentafluorobenzene sulfonyl fluorescein (Cayman Chemical) (Candel et al. 2014; de Oliveira et al. 2015). About 72 hpf embryos were collected in a 96‐well plate with 50 μM of the substrate in 1% DMSO for 1 h. The mean intensity of fluorescence was determined using *ImageJ* software, with a region of interest placed in the tail to quantify H₂O₂ production.

### 2.9 Acridine Orange (AO) Staining Assay

Cell death of 3 dpf larvae was measured by acridine orange (AO) staining as described in the *ZFIN Protocol Wiki* (https://zfin.atlassian.net/wiki/spaces/prot/overview). After staining, the larvae were transferred to a Petri dish containing buffered tricaine (200 μg/mL) dissolved in E3 and imaged using a fluorescence magnifying glass (Leica M205 FCA) equipped with a digital camera (Leica DFC 365 FX) and set up with a green, fluorescent filter. The images were analyzed to quantify the number of AO^+^ cells.

### 2.10 Survival Curves

Zebrafish larvae were monitored every 24 h for clinical signs of disease and mortality under a magnifying glass (Leica M150C) equipped with a digital camera (DMC 5400, Leica).

### 2.11 Drug Treatment and Imaging

At 24 hpf, ST2 larvae were treated with resveratrol (10 μM), navitoclax (10 μM), or vehicle alone (DMSO 0.1%) for 2 days (Figure 4A). On the third day post‐fertilization (3 dpf), the larvae were sampled and/or imaged by using a fluorescence magnifying glass (Leica M205 FA) equipped with a digital camera (Leica DFC 365 FX). To generate survival curves, 48 hpf ST2 larvae were treated with resveratrol (10 μM), navitoclax (10 μM), or vehicle alone (DMSO 0.1%) and compounds renewed every 2 days (Figure 4I).

### 2.12 Statistical Analysis

**FIGURE 2:**
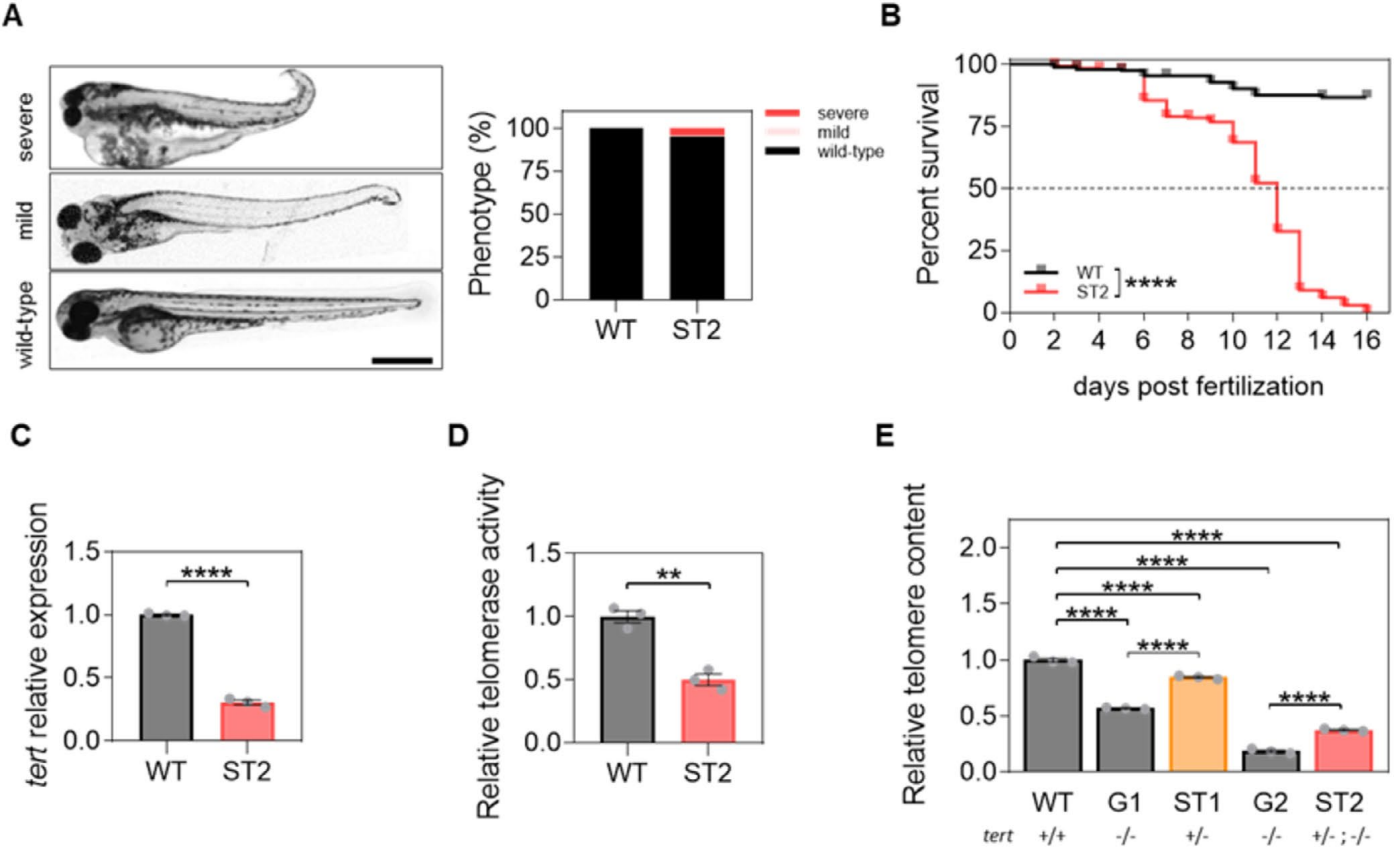
Phenotype and telomeric characterization of the ST2 model. (A) Representative images and classification of 48 hpf larvae by phenotype. (B) Kaplan–Meier plot of survival of WT (*n* = 273) and ST2 larvae (*n* = 615). The graph shows the accumulation of 5–10 independent experiments. (C) The mRNA level of *tert* was determined in 3 dpf zebrafish larvae by real‐time RT‐qPCR, normalized against *rps11*. (D) Telomerase activity was measured quantitatively in 3 dpf larvae by qTRAP using 0.1 μg of protein extract. (E) Telomere length was measured in 3 dpf larvae by qPCR using 16 ng of gDNA and determined as the telomere content normalized to the single‐copy gene *rps11*. Bars represent the mean ± SEM of 3 independent experiments (*N* = 3, represented by dots) of 25 pooled larvae (*n* = 25), and relative to WT group. In A, differences are not statistically significant according to two‐way ANOVA followed by Sidak's multiple comparison test. **p < 0.01; ****p < 0.0001, according to the log rank test (B), unpaired *t*‐test (C, D) and one‐way ANOVA followed by Holm‐Sidak's multiple comparison test (E). Scale bar: 500 μm.

**FIGURE 3:**
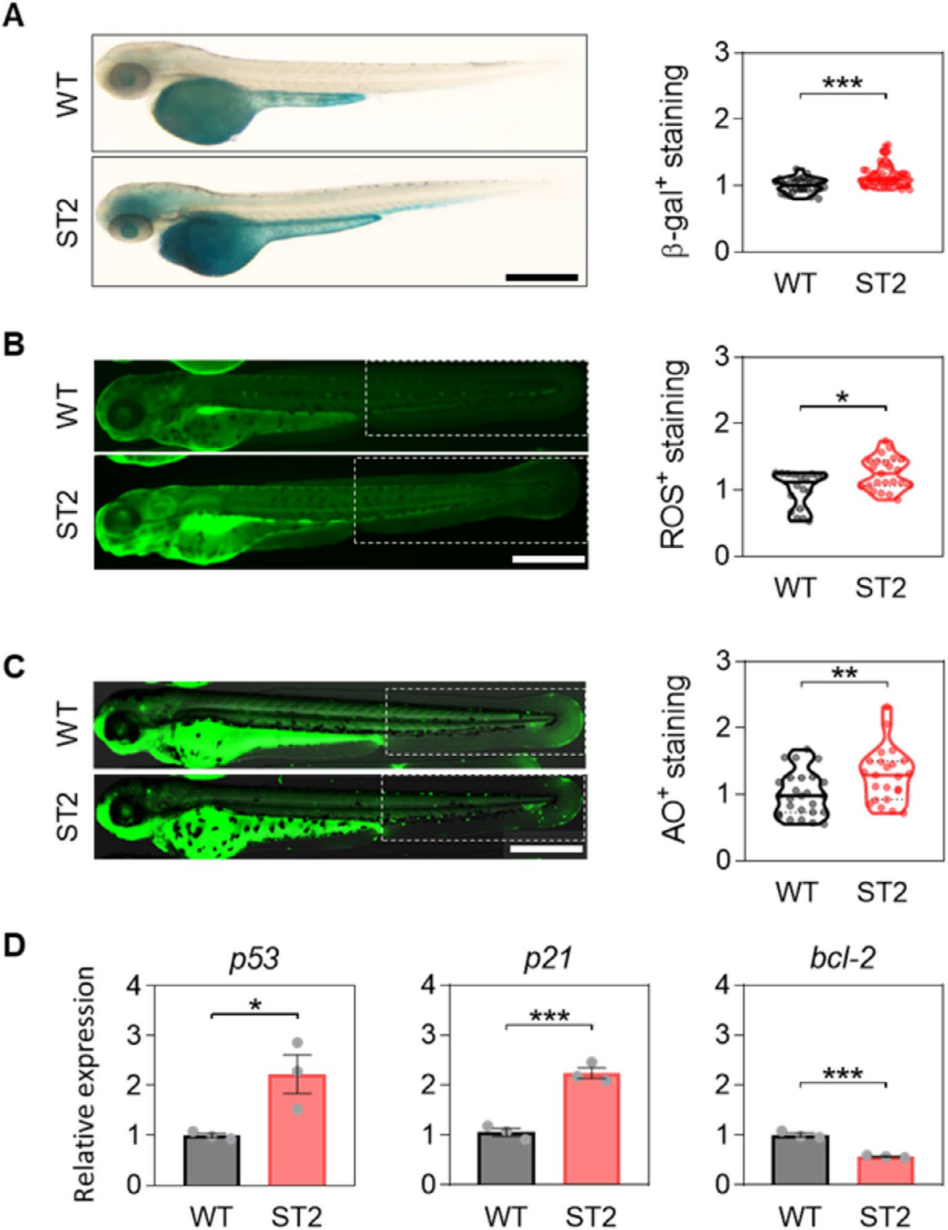
The ST2 model shows increased cellular senescence, apoptosis and premature death. (A) Representative images of β‐galactosidase (β‐gal) staining of 3 dpf WT and ST2 larvae and quantification of the cellular senescence levels. (B) Representative images of reactive oxygen species (ROS) staining and quantification of the cellular oxidative stress levels in 3 dpf WT and ST2 larvae. (C) Representative images of acridine orange (AO) staining and quantification of the DNA fragmentation levels in 3 dpf WT and ST2 larvae. The discontinuous white square represents the ROI for quantification. Violin plots with the media represented as a horizontal line show the distribution of β‐gal^+^ (A), ROS^+^ (B) and AO^+^ (C) staining relative to WT. The violin plots are overlaid with the raw data, where each point represents an individual. (D) The mRNA levels of apoptosis‐related genes were determined by real‐time RT‐qPCR and normalized to *rps11* in 3 dpf zebrafish larvae. Bars represent the mean ± SEM of 3 independent experiments (*N* = 3, represented by dots) of 25 pooled larvae (*n* = 25), and relative to WT. *p < 0.05; ***p < 0.001; ****p < 0.0001, according to Mann–Whitney test and unpaired *t*‐test (C, D). ROI: Region of interest. Scale bar: 500 μm.

**FIGURE 4:**
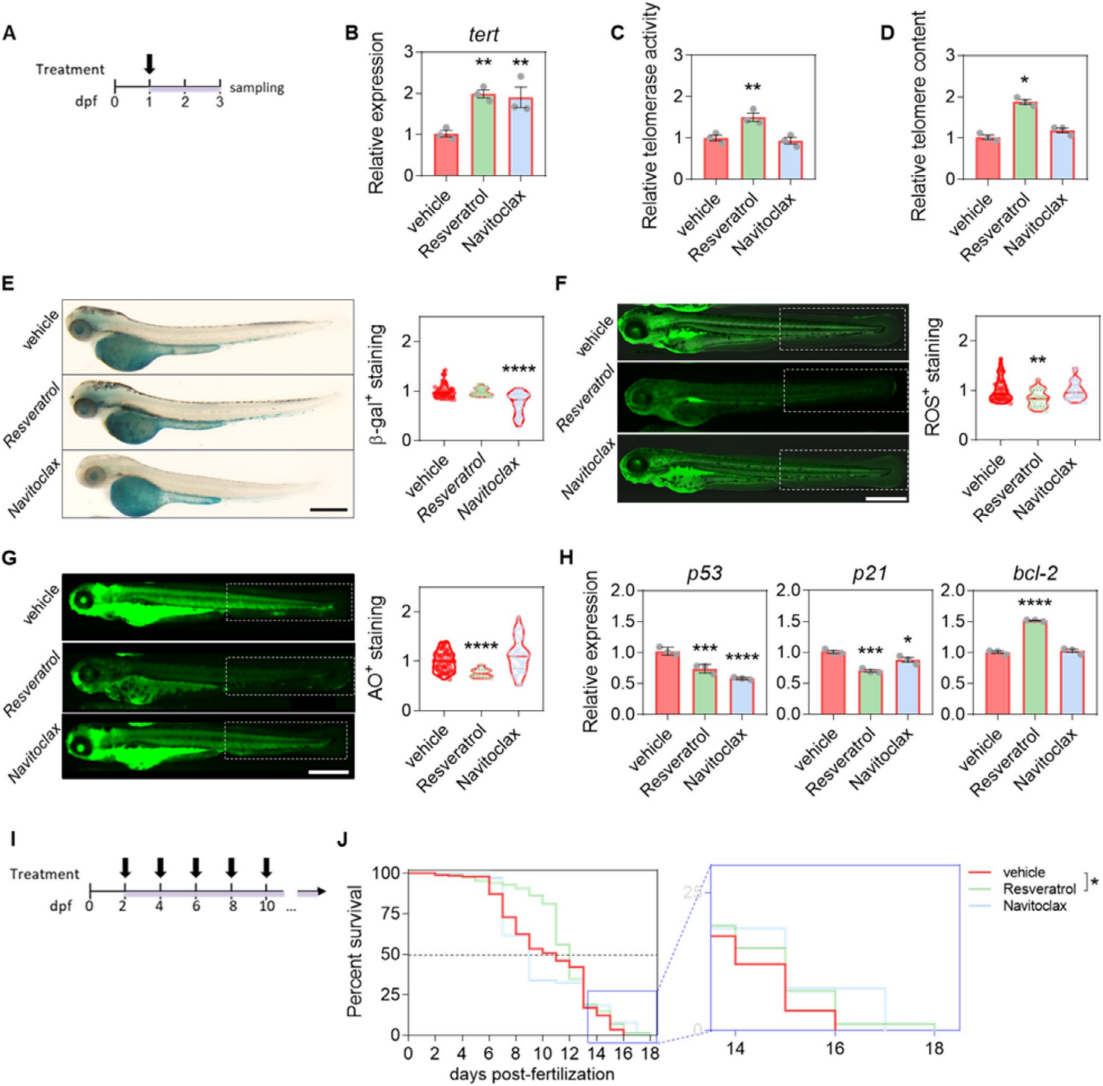
Evaluation of the antiaging effect of resveratrol and navitoclax in the ST2 model. (A) Workflow to study the antiaging effect of the treatment. (B) The mRNA level of *tert* was determined in 3 dpf ST2 larvae treated with resveratrol or navitoclax by real‐time RT‐qPCR and normalized against *rps11*. (C) Relative telomerase activity was measured quantitatively in 3 dpf zebrafish larvae treated with resveratrol or navitoclax by qTRAP using 0.1 μg of protein extract. (D) Telomere length was measured in 3 dpf larvae treated with resveratrol or navitoclax by qPCR using 16 ng of gDNA and determined as the telomere content relative to the single‐copy gene *rps11*. Bars show the mean ± SEM of 3 independent experiments (*N* = 3, represented by dots) of 25 pooled larvae (*n* = 25), and relative to vehicle. (E) Representative images and quantification of the cellular senescence levels by β‐galactosidase (β‐gal) staining assay in 3 dpf ST2 larvae after treatment, where β‐gal^+^ cells are stained in blue. (F) Representative images of reactive oxygen species (ROS) staining and quantification of the cellular oxidative stress levels in 3 dpf ST2 larvae after treatment, where ROS^+^ cells are stained in green. (G) Representative images and quantification of the cellular death levels by acridine orange (AO) staining assay in 3 dpf ST2 larvae after treatment, where AO^+^ cells are stained in green. In (E–G), the discontinuous white square represents the ROI for quantification. Violin plots with the media shown as a horizontal line show the distribution of β‐gal^+^ (E), ROS^+^ (F) and AO^+^ (G) staining relative to vehicle. Violin plots are overlaid with the raw data, where each point represents an individual. (H) The mRNA levels of apoptosis‐related genes were determined by real‐time RT‐qPCR and normalized to *rps11* in 3 dpf ST2 larvae after treatment. Bars represent the mean ± SEM of 3 independent experiments (*N* = 3, represented by dots) of 25 pooled larvae (*n* = 25) and relative to vehicle. (I) Workflow to study the effect of the treatment, renewed every 2 days, on the survival of ST2 larvae. (J) Kaplan–Meier plot of the survival of vehicle (*n = 275*), resveratrol (*n* = 168), and navitoclax (*n* = 65) treated ST2 larvae. The dashed line represents the half‐life. On the right is the magnification of the last days of survival is shown. The graph shows the accumulation of 3–5 independent experiments. *p < 0.05; **p < 0.001; ***p < 0.001; ****p < 0.0001, according to one‐way ANOVA followed by uncorrected Fisher's LSD multiple comparison test (B, C, H), Kruskal–Wallis followed by uncorrected Dunn's multiple comparison test (D–F), Brown–Forsythe and Welch ANOVA test followed by *t*‐test with Welch's correction multiple comparison test (G) and log rank test (J). ROI: Region of interest. Scale bar: 500 μm.

Statistical analysis was performed by using GraphPad Prism 8. Results are presented as mean ± SEM, unless otherwise stated. Differences were considered significant when *p* < 0.05. Differences between two samples were analyzed by parametric or nonparametric test. Data from more than two samples were analyzed by analysis of variance. Survival curves were analyzed by log‐rank (Mantel‐Cox) test (see (Figures 1, 2, 3, 4) legends for further details).

## 3 Results

### 3.1 A New Model of Rapid Aging Zebrafish With Reduced Telomerase Activity, Shortened Telomeres and Premature Death

We obtained a shortened telomere zebrafish model to study aging and rejuvenation at the larval stage, derived from the Tert‐deficient model (*tert* ^−/−^) and corresponding to the second round of Tert‐haploinsufficient (*tert* ^+/−^) zebrafish crosses, taking the necessary precautions to avoid progressive telomere shortening in the tert^+/−^ (P) used for the crosses (Figure 1) (our results and (Henriques and Ferreira 2024)). Although the progeny exhibited a combination of heterozygous and knockout genotypes, after 48 h post fertilization (hpf), the majority displayed a wild‐type phenotype (94.9%), while the remainder displayed a mild (0.85%) or severe (4.24%) phenotype (Figure 2A). Evaluation of life expectancy using Kaplan–Meier survival curves showed that ST2 *z*ebrafish were unable to survive the larval stage (Figure 2B). This premature death can be explained from the telomeric point of view, since at 3 days post fertilization (dpf) the larvae showed a significant reduction in *tert* mRNA levels (Figure 2C). As a consequence, telomerase activity was also significantly reduced (Figure 2D), which translated into a significant reduction in telomere content (Figure 2E) in accordance with the anticipated progression (Figure 1, bottom). This is the reason why this particular model has been designated ST2 (Shortened Telomere, 2nd round).

### 3.2 The ST2 Larvae Show Increased Cellular Senescence and Apoptosis

Since telomere attrition leads to cellular senescence and cellular senescence can trigger premature aging, we determined the level of cellular senescence in the ST2 model by measuring the senescence‐associated β‐galactosidase (SA β‐gal) biomarker in 3 dpf larvae. Whole larval β‐gal staining revealed a high level of cellular senescence compared to the control (Figure 3A). As the increase in senescence also affects the mitochondria, this was reflected by an increase in ROS production in the ST2 model (Figure 3B). On the other hand, since both critically short telomeres and increased ROS induce apoptosis, we performed an acridine orange assay in the ST2 model and found an increased level of cell death in the ST2 larvae compared to the control (Figure 3C). We also examined the gene expression profile of the proapoptotic genes *p53* and *p21*, and the antiapoptotic gene *bcl‐2*. In our ST2 model, the expression of both *p53* and *p21* was induced, while the expression of *bcl‐2* was inhibited compared to the control, supporting the previous results (Figure 3D). Taking together, these results validate the ST2 model as an excellent model of premature larval aging with a phenotype compatible with rapid drug screening.

### 3.3 Evaluation of the Antiaging Potential of Resveratrol and Navitoclax in the ST2 Model

Having confirmed the premature aging phenotype in the ST2 larval model, we decided to perform a proof of concept with two reference molecules, resveratrol and navitoclax. They represent polyphenols and senolytics, respectively, as both groups have been extensively studied and proposed to treat aging (Kirkland and Tchkonia 2020; Shen et al. 2017).

In the context of telomere biology, both resveratrol and navitoclax treatment resulted in an increase in *tert* mRNA levels (Figure 4B). However, only resveratrol was able to induce telomerase activity (Figure 4C) and, consequently, to increase the telomere content (Figure 4D). Regarding cellular senescence, only navitoclax was able to reduce the senescence‐associated β‐gal‐positive area compared to untreated ST2 larvae (Figure 4E), and the decline in the number of senescent cells is reflected indirectly in a reduction in the expression of proapoptotic genes (Figure 4H). The effect of resveratrol on the telomere content was reflected directly in the level of oxidative stress and cell death, with a significant reduction of ROS and DNA fragmentation levels, while navitoclax had no effect (Figure 4F,G). As a direct consequence, the treatment with resveratrol caused a switch in the apoptosis‐related gene expression profile, reducing the expression of the proapoptotic genes *p53* and *p21*, and increasing that of the antiapoptotic gene *bcl2* (Figure 4H). Finally, we evaluated the effect of resveratrol and navitoclax on the lifespan of ST2 larvae. Treatment with resveratrol, but not navitoclax, increased the half‐life of ST2 larvae by 5% and survival by 12.5% (Figure 4J).

## 4 Discussion

Advances in both public health and medicine have increased life expectancy, but have also increased susceptibility to many age‐related diseases, which is a major public health problem from a social and economic perspective (Melzer et al. 2020). There is currently great interest in understanding the basic mechanisms that drive aging and the relationship between aging and age‐related chronic diseases in order to slow or delay their onset and thus increase healthy life expectancy (Kennedy et al. 2014).

Animal models are essential in the study of aging, both for the study of diseases at the organismal level and for drug development and repurposing. Due to its characteristics, the zebrafish is an excellent model to study aging (Carneiro, de Castro, et al. 2016). There are several models that allow us to approach the study of SIPS caused by immunodeficiency (Novoa et al. 2019), chronic inflammation (Hernandez‐Silva et al. 2022) or DNA damage (Morsli et al. 2023). However, the telomerase‐deficient premature aging model for studying replicative senescence is limited by life expectancy and phenotype. While lifespan is still too long in the telomerase‐deficient first generation (*tert* ^−/−^ G1) (Anchelin et al. 2013; Henriques et al. 2013; Henriques and Ferreira 2024), the second generation (*tert* ^−/−^ G2) is so affected that almost 70% of larvae die in the first 24 h (Anchelin et al. 2013). It is therefore of great interest to obtain models of rapid aging at a young age.

Crucially, the reintroduction of telomerase reverses telomere shortening and promotes longevity in the zebrafish model (Anchelin et al. 2013). Consequently, we have established a larval model of shortened telomere‐induced premature aging, termed the ST2 model (Figure 1), achieved by crossing *tert* ^+/−^ fish with the Tert‐deficient zebrafish line. This enhancement significantly improves survival rates, allowing research to be carried out at the larval stage, thus overcoming the main limitations of the *tert* ^−/−^ G2 model. First, the phenotype of the ST2 larvae is predominantly wild type (95%) (Figure 2A), compared to barely 10% in the *tert* ^−/−^ G2 model (Anchelin et al. 2013). ST2 larvae showed the main features of shortened telomere‐induced premature aging: (i) reduced *tert* expression; (ii) reduced telomerase activity; and (iii) telomere attrition (Figure 2C–E) and premature death at the larval stage (Figure 2B). Due to telomere shortening, we also observed an accumulation of senescent cells and an increase in oxidative stress levels in ST2 larvae compared to their wild‐type counterpart (Figure 3A,B). Relatedly, the ST2 model showed a higher level of cell death, as determined by both DNA fragmentation levels and the quantification of *mRNA* levels of several genes related to cell cycle arrest such as *p53* and *p21*, or cell survival, such as *bcl‐2* (Figure 3C,D). To increase healthy life expectancy, the search for molecules with antiaging potential is necessary in order to reduce the burden of age‐related diseases and disabilities (Kennedy et al. 2014). Zebrafish larvae have emerged as an undisputed model due to their ease, cost‐effectiveness, and speed in high‐throughput screening of compounds. Recently, Morsli and colleagues have described a DNA damage model that allows the evaluation of senolytics in a 5‐day assay (Morsli et al. 2023). However, this model is difficult to use because it requires the use of ionizing radiation to induce DNA damage. In contrast, the model described here is a shortened telomere‐induced premature aging model that does not require any external stimulus. Furthermore, it is important to highlight that the ST2 progeny is a combination of Tert knockouts and heterozygotes, which enhances its utility as a model for antiaging screening. Although the aging effects are not as severe as in G2, which could hamper certain studies, they are significant enough to result in a lifespan of only 2 weeks. Furthermore, the heterogeneity of the genotypes can prove beneficial in screening, allowing the identification of two categories of rejuvenating compounds: those capable of increasing telomere length by activating *tert* expression, and those that do so independently of telomerase or telomere length. Following an initial rapid screening in the ST2 model, the subsequent step is the validation of the results in models with a single genotype for the characterization of the mechanism, which may be either independent or dependent on Tert.

Dyskeratosis congenita (DC) is associated with mutations in genes encoding proteins of the telomerase complex or telomere‐binding proteins (Savage 2022). However, the utility of the ST2 model may be limited, as these mutations may not directly affect telomere length (Gutlapalli et al. 2020) in patients with alterations in other components of the telomerase complex. These mutations can lead to a significant loss of function or indirectly impair telomerase activity. Nevertheless, such patients may benefit from telomere length elongation through alternative mechanisms, as the ST2 model can identify both Tert‐dependent and ‐independent compounds, providing a versatile approach for developing targeted therapies for different types of telomeropathies. However, it is crucial to complement the ST2 model with other models that accurately reflect specific mutations, such as those affecting Dyskerin (Balogh et al. 2020).

As a proof of concept, we decided to evaluate the effects of two reference molecules, resveratrol, and navitoclax. In terms of telomere biology, resveratrol treatment increased *tert* mRNA levels, telomerase activity, and telomere length. However, the induction of *tert* expression produced by navitoclax treatment had no effect on telomerase activity or telomere length (Figure 4B–D). As anticipated, navitoclax demonstrated the capacity to diminish the number of senescence‐associated β‐gal‐positive cells in comparison to the untreated ST2 larvae (Figure 4E), thereby lowering the mRNA levels of proapoptotic genes (Figure 4H). Nevertheless, no notable impact was discerned with respect to ROS or DNA fragmentation levels (Figure 4F,G). In response to the effect on telomere length, a significant reduction in ROS and DNA fragmentation levels was observed following resveratrol treatment (Figure 4F,G). Additionally, a switch in the apoptosis‐related gene expression profile was noted (Figure 4H), which ultimately translated into a 5% increase in the half‐life of ST2 larvae and a 12.5% increase in survival (Figure 4J). However, treatment with navitoclax had no positive impact on the survival of ST2 larvae. Given that short telomere length represents the limiting factor for the survival of ST2 larvae, the reversal of cellular senescence with senolytics proved to be an ineffective strategy.

In this study, we validate the ST2 zebrafish larvae model as an exceptional tool for both drug screening and aging research, particularly given the rapid organogenesis that occurs in larvae. The ST2 model represents a vertebrate system that facilitates the rapid identification of molecules with antiaging potential, addressing the needs of an increasingly aging society that seeks to enhance health and longevity as soon as possible.

## Author Contributions

**Methodology:** David Hernández‐Silva, María D. López‐Abellán, Francisco J. Martínez‐Navarro, Jesús García‐Castillo, Francisca Alcaraz‐Pérez; **Formal analysis:** David Hernández‐Silva, Jesús García‐Castillo, María L. Cayuela, Francisca Alcaraz‐Pérez; **writing original draft:** David Hernández‐Silva, Jesús García‐Castillo, María L. Cayuela, Francisca Alcaraz‐Pérez; **conceptualization:** María L. Cayuela, Francisca Alcaraz‐Pérez; **funding adquisition:** María L. Cayuela.

## Conflicts of Interest

The authors declare no conflicts of interest.

## Supporting information


**Table S1.** Primers used in this study.

## Data Availability

The data that support the findings of this study are available upon reasonable request.
