# Supplementary material for: Development of a Short Telomere Zebrafish Model for Accelerated Aging Research and Antiaging Drug Screening
Source: Aging Cell. 2025 Feb 8;24(6):e70007. doi: 10.1111/acel.70007 (PMC12151894; doi:10.1111/acel.70007)
Supplement: Supplementary file 1 — Table S1. Primers used in this study. [file ACEL-24-e70007-s001.pptx]

## Slide 1
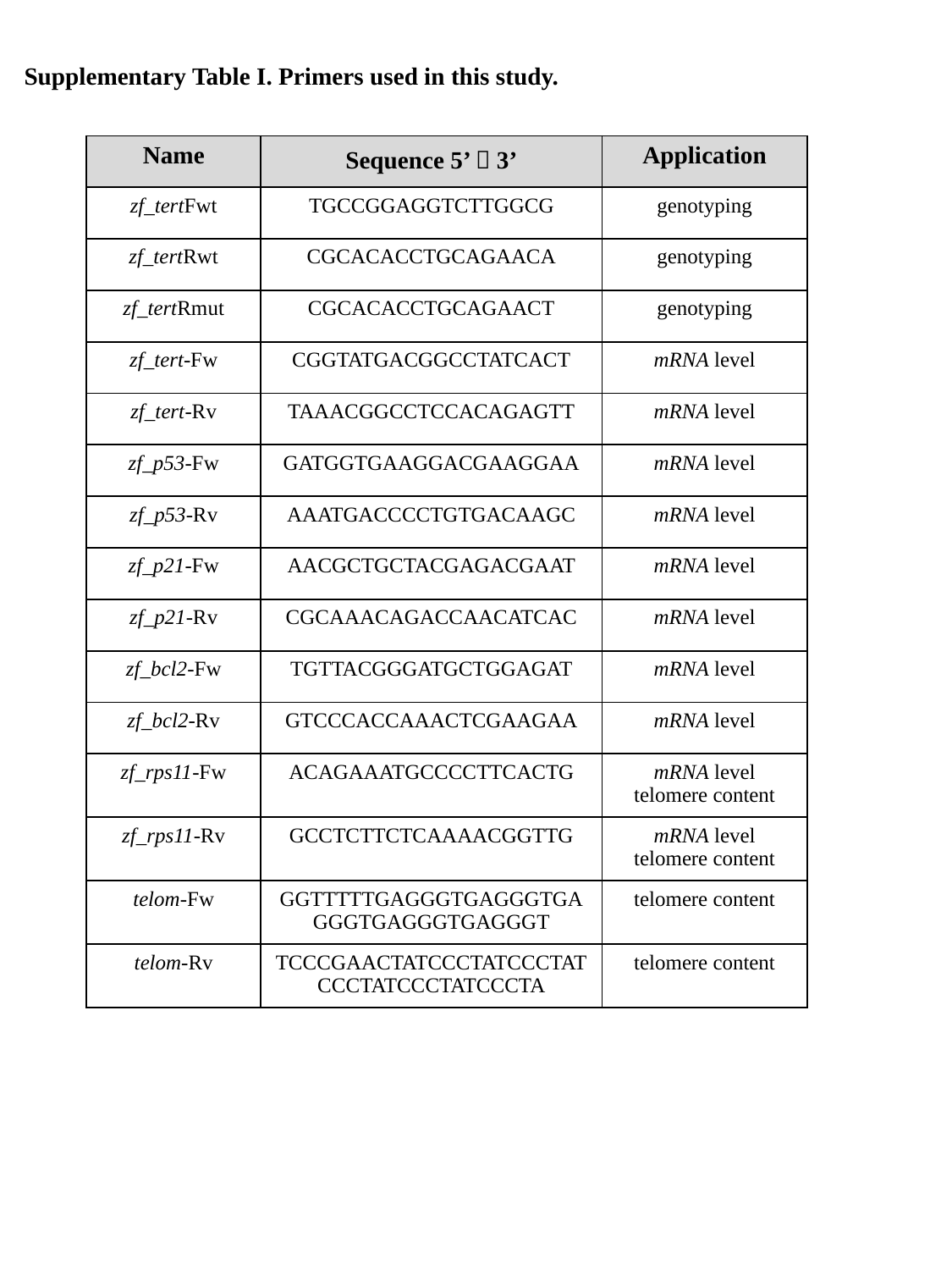

Supplementary Table I. Primers used in this study.
| Name | Sequence 5’  3’ | Application |
| --- | --- | --- |
| zf\_tertFwt | TGCCGGAGGTCTTGGCG | genotyping |
| zf\_tertRwt | CGCACACCTGCAGAACA | genotyping |
| zf\_tertRmut | CGCACACCTGCAGAACT | genotyping |
| zf\_tert-Fw | CGGTATGACGGCCTATCACT | mRNA level |
| zf\_tert-Rv | TAAACGGCCTCCACAGAGTT | mRNA level |
| zf\_p53-Fw | GATGGTGAAGGACGAAGGAA | mRNA level |
| zf\_p53-Rv | AAATGACCCCTGTGACAAGC | mRNA level |
| zf\_p21-Fw | AACGCTGCTACGAGACGAAT | mRNA level |
| zf\_p21-Rv | CGCAAACAGACCAACATCAC | mRNA level |
| zf\_bcl2-Fw | TGTTACGGGATGCTGGAGAT | mRNA level |
| zf\_bcl2-Rv | GTCCCACCAAACTCGAAGAA | mRNA level |
| zf\_rps11-Fw | ACAGAAATGCCCCTTCACTG | mRNA level telomere content |
| zf\_rps11-Rv | GCCTCTTCTCAAAACGGTTG | mRNA level telomere content |
| telom-Fw | GGTTTTTGAGGGTGAGGGTGAGGGTGAGGGTGAGGGT | telomere content |
| telom-Rv | TCCCGAACTATCCCTATCCCTATCCCTATCCCTATCCCTA | telomere content |
